# Numerical investigation of a squeezing flow between concentric cylinders under the variable magnetic field of intensity

**DOI:** 10.1038/s41598-022-13050-2

**Published:** 2022-06-01

**Authors:** Shabnam, Sun Mei, Muhammad Sohail Khan, Omar Mahmoud, Ahmed M. Galal

**Affiliations:** 1 School of Mathematical Sciences, Jiangsu University, Zhenjiang, 212013 Jiangsu China; 2 Petroleum Engineering, Faculty of Engineering and Technology, Future University in Egypt, New Cairo, 11835 Egypt; 3 Mechanical Engineering Department, College of Engineering, Prince Sattam Bin Abdulaziz University, Wadi ad-Dawasir, 11991 Saudi Arabia; 4 Production Engineering and Mechanical Design Department, Faculty of Engineering, Mansoura University, P.O. 35516 Mansoura, Egypt

**Keywords:** Applied mathematics, Computational science

## Abstract

The ongoing research aims to examine the mass and heat transmission phenomena of squeezing flow between two concentric cylinders under the effect of heat sources and magnetic fields. The impacts of the Lorentz force on the behavior of the liquid flow are elucidated via a magnetic field incorporated in the momentum equation. Furthermore, within concentric cylinders, the expression $Q = \frac{Q_0}{1-\beta t}$ has been employed as a source/sink. The proposed model of PDEs formulates the physical phenomena of time-dependent incompressible two-dimensional squeezing flow via modified Navier-Stokes equation, energy equation, and mass transfer equation, and variable magnetic field. The proposed model involved a highly nonlinear system of PDEs, which has been reduced into a system of ODEs via Lie group of similarity transformation and subsequently solved numerically in MATLAB by Parametric Continuation Method. The direct impact of the squeezing parameter on the profile of temperature and concentration has been observed. The results shown that an increment in the heat source indicates a decline in the liquid temperature profile, that an increment in the heat source indicates a decline in the liquid temperature profile. An increment in the heat source indicates a decline in the liquid temperature prof. At the same time, an inverse relationship is observed for the concentration profile. Therefore, we have witnessed a significant increase in the velocity profiles of the flow, mainly as a result of the heat absorption coefficient. In addition, the declining effect of the Soret number on the concentration profile is noticed. It has been found that it enhanced the entropy generation rate for *Pr*, $\Omega$, and *Ec*, while an opposite impact has been noticed at the Bejan number. The numerical outcomes of the proposed model that explain fluid flow characteristics and fluid flow characteristics are quantitatively elucidated by tables and displayed graphically. The comparison of two numerical results in the cases are found to be in good agreement, as shown in Tables.

## Introduction

Work on the movement of liquids with stretching cylinders has attracted the attention of many researchers. It should be noted that the flow of the boundary layer due to surface shrinkage or stretching is a related type of flow that is reflected in several engineering and industrial processes. There are many uses of such flow in the engineering process, for instance, in the polymer and metallurgy process such as extraction and manufacturing of polymer and rubber sheets, melting, hot rolling, paper manufacturing, wire drawing, and glass fiber manufacturing.

It is noteworthy that the unsteady nature of many liquid movements plays a more practical role and has gained considerable attention in recent years. In several applications, the ideal liquid flow problems around the instrument is not usually steady, but the undesirable volatile effects are either due to the body’s own involvement or due to fluctuations or inconsistencies in the surrounding fluid. Otherwise, some types of equipment require the time-dependent movement of fluid to perform their basic functions has been analyzed by McCroskey^1^. The work on the time-dependent boundary layers is important because all boundary layers, which exist in practice, are in a sense, time-dependent. The time-dependent viscous fluid movement has been extensively studied, and all the features of transient impacts are now less or more familiar to the researcher in mechanics. Wang^2^ analyzed the incompressible steady viscous liquid movement outside the stretching hollow cylinder while the fluid was not flowing. The same problem has been expanded by incorporating the effects of suction and injection by Ishaketal^3^. Talion is^4^, Wang^5^ and Riley^6^ have summarized the basic ideas and significant contributions to the subject. A better interpretation of unsteady liquid flow and applying this approach to newly designed methods provide significant enhancements in the performance, reliability, and price of several fluid dynamic equipment. McLaughlin and Wang^7^ seem to have been the first in the field to study the fluid flow at a shrinking surface. To adjust this fluid flow, the liquid is pushed in the slot direction and the fluid flow is completely unlike the stretching case. These authors have shown that massive suction is usually required to maintain flow on a shrinking sheet. Another instance of fluid moving towards a shrinking sheet is the incrementing shrinkage done by Wang^8^. It has been reported that injection reduces the rate of heat transfer and skin friction to the surface, whereas suction works in the opposite direction.

In the recent past, Fang et al.^9^ investigated the time-dependent viscous fluid movement on an extended stretching cylinder that gives the exact solution of the Navier-Stokes equation. They pointed out that due to the cylinder’s expansion, there is a reverse flow, and the flow field is firmly affected by the Reynolds number and unsteady parameter. Later on, Fang et al.^10^ studied the time-dependent viscous fluid flow numerically in the outside region of the extending or contracting type cylinder. Stefan^11^ presented his work on squeezing fluid flow under the lubricating approximation approach. Domain and Aziz^12^ have analyzed the impacts and properties of a squeezing flow of viscous liquid under the magnetic field between two parallel discs. Siddiqui et al.^13^ investigated the impact of hydro-magnetic squeezing flow of the thick liquid between two long parallel plates. The models were solved using the Homotopy Perturbation Method (HPM) in both of these studies. likewise, Rashdi et al.^14^ studied the hydrodynamic of the viscous squeezing fluid flow using Homotopy Analysis Method (HAM).

The unsteady/steady electroosmotic liquid motion through an infinite expanded cylindrical channel of diameters 10 to 100 nm has been investigated by Nayak^15^. They have used a combination of (NaCl + H2O) for numerical computation of mass, velocity, potential, and mixing efficiency, the outcomes are acquired in steady and unsteady cases for small, large, or equal to the outcomes developed in steady and unsteady cases for small, large, or equal to electric double layer (EDL). Likewise, Shekholeslami et al.^16^ analyzed the behavior of time-dependent nanofluid flow between two parallel squeezing plates, solved the proposed model by (ADM), and concluded that the Nusselt number increments with the increment in the volume fraction and Eckert number. In contrast, it reduces with the enhancement of the squeezing number. Rajvanshi et al.^17^ used the Brinkman model to investigate the MHD squeezing flow of viscous non-compressible liquid in a porous medium between two permeable revolving plates. Sangapatnam et al.^18^ studied the radiation and mass transfer impact on the MHD flow past natural convection through iso-thermal vertical plate under viscous dissipation. The effect of radiation, MHD, and mass transfer on the transient flow of natural convection through hot vertical porous plates in the presence of viscous dissipation was analyzed by Prasad and Reddy^19^. Rajvanshi et al.^20^ studied the incompressible viscous squeezing fluid flow between two highly absorbent revolving plates in a porous medium. Solutions are acquired for relatively low density optically thin mediums, and are suitable for liquids with a thickness much higher than wall roughness.

Pattnaik et al.^21^ analyzed the coupled effects of convective heating, Ohmic electro thermal dissipation, and exponential heat source on magnetized micropolar nanofluid on an expanded plate by shooting quadrature. The comprehensive impacts of conjugate wall conduction and convective boundary heating on the flow of magnetized thermo-solute layer from the nonlinear long container with hydrodynamic thermal concentration slip and radioactive heat transfer were analyzed by Uddin et al.^22^. Hosseinzadeh et al.^23^ performed the hydrothermal assessment with the conventional fluid of ethylene glycol-water (50%-50%) consistingof hybrid nanomaterials (TiO2-MoS2) in an octagon with the elliptical cavity in the center. Their outcomes showed that with an increment in the Rayleigh number from 10 to 100, the mean Nusselt number improved by about (61.82%). The flow of hybrid nanofluid obtained by the combination of ethylene glycol-water (50%-50%) with nanomaterials (MWCNT-Ag) at a vertical stretching cylinder was studied by Hosseinzadeh et al.^24^. The outcomes indicated that the spherical ana lamina geometries of nanomaterials have larger variation, and lamina gives (6%) smaller value. They also show that with rising Hartman numbers, the radial velocity for hybrid nanomaterials decreased by (9.68%). Marathi et al.^25^ analyzed the influence of magnetic nanomaterials in incrementingheat transport in a tribological system under connective type heating boundary conditions (Robin). Rehan et al.^26–28^ studied the augmented viscosity pattern and heat transfer behavior of a transient two-dimensional non-compressible squeezing flow of ion-nano-fluid between two long parallel concentric cylinders. To check heat transfer capacity, three different nano components like Titanium oxide, Copper, and Aluminum of volume fraction from 0.1 to 0.7 nm are suspended into an ionic fluid in turns. The Maxwell Garnet model of thermal conductivity andthe Brinkman viscosity model of nanomaterials have been adopted.

Philip et al.^29^, studied the influence of slip velocity, magnetic field, and concentration for an unsteady fluid flow and heat transfer on parallel plates. For its numerical results, they have used Range-Kutta of order (four-five) in the frame of shooting techniques which produced interesting results for the following parameters as Schmidt number, Hartman number, Nusselt number, velocity and concentration on Sherwood number, velocity slip parameter, Skin friction, squeeze number and volume fraction of nanoparticle on temperature. Further, Siddiqui et al.^30^ studied the hydromagnetic influences of a viscous fluid in horizontal parallel plates, and solves the proposed model by HPM. Sheikholeslami et al.^31^the numerical scheme then solved interesting results and the proposed model investigated the effect of forced convection heat transfer and nano-uniform magnetic field on nanofluid and found interesting results. The numerical schemethe numerical scheme then solved the proposed model solved interesting developments and the proposed model. Further, Sheikholeslami et al.^32^ studied the nanofluid flow in a two-stage simulation with heat transfer on parallel plates, and solves the constructed model by HPM. Next, Sheikholeslami and Ganji^33^, examined the provision of mass transfer and heat transfer in a parallel channel on unsteady nanofluid in the viscous dissipation existence and the effect of radiation. A group,Okango et al.^34^, studied Hall?s current effect in the presence of variable magnetic fields in the vertical porous flat plates and the flow between them. The flow is steady and the selected domain is laminar, but the whole system rotates with a uniform angular velocity around the normal axis of the plate. Bejan^35,36^, suggested in their proposed model that the flow of variable parameters can be taken to reduce the Irreversibility in the heat transfer process through specific convective. Hijleh et al.^37^, observed through a rotating cylinder the laminar mixed convective of the entropy and concluded that the increase in buoyancy parameter and Reynolds number occur due to the rise of entropy generation. Tasnim et al.^38^, reviewed the study of the first law and second law of thermodynamics regarding the flow properties and heat transfer in a magnetic field through two parallel vertical plates with a porous medium. Odat et al.^39^, studied the effects of entropy generation through the laminar flow past a flat plate in a magnetic field. They noticed that the magnetic field intensity leads to an increased entropy generation rate.

We can readily review from the literature as mentioned earlier that Rehan et al.^26^ examined the impact of a changeable magnetic field in a tribological process between two concentric cylinders regarding the enhancement of heat and mass transfer. The method that has been scrutinized comprises the transmission of Newtonian magnetic lubricant between two concentric cylinders under the influence of magnetism. The present study is about the entropy investigation of two-dimensional fluid movement and the heat transfer with the coupled Lorentz force in the presence of a heat sink/source. The numerical outcomes of different emerging parameters like heat source *Hs*, Schmidt number *Sc*, Prandtl number *Pr*, Magnetic parameter *M*, Bejan number *Be*, squeezing number *S*, magnetic field, skin friction coefficient, Nusselt number, and entropy production are computed and subsequently displayed through various tables and graphs. The proposed fluid flow model has several applications in the field of transport, biomedical, industries, and electronics.

We have mainly focused on the following objectives, To developed and analyzed mathematical model for an unsteady flow and incompressible flow on two concentric cylinders with variable magnetic ?eld.

To investigated physical properties such as skin friction, velocity field, magnetic field, temperature, concentric distribution, entropy generation and Bejan number.

To reduce skin friction, to control flow speed and to enhance temperature distribution. Thought this analysis the model partial differential equations is transformed to ordinary differential equations and has analyzed numerically through parametric continuation method. For comparison results is also be calculated with the help of BVP4C package.

**Figure 1:**
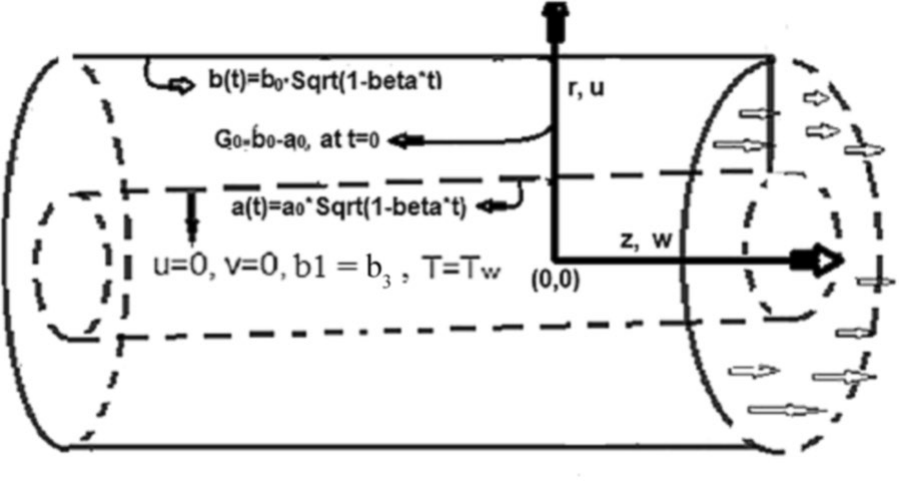
Geometry of the problem with Coordinate system.

## Formulation

We consider time-dependent two-dimensional laminar liquid boundary layer flow between two concentric cylinders in the presence of a variable magnetic field to analyze heat transfer behavior. The liquid updated in the positive *z*-direction is presumed to be infinite. The coordinate system in the polar form (*r*, 0, *z*), has been taken at the centerof the inside cylinder, and hence the velocity components *u* and *w* lie along the direction of *r*-axis and *z*-axis, respectively, as displayed in Fig. 1. It is presumed that the diameters of the concentric cylinder dependent on time, and its time-dependent radii $a(t) = \sqrt{1-\beta t}$ and $b(t) = \sqrt{1-\beta t}$ of the inside and outside cylinders respectively , where *b* represents the extension/contraction power, *t* shows time, and $a_0$ shows radius of the inside cylinder at $t = 0$. The liquid is assumed to be a symmetric flow with fixed viscosity $\mu$, and density $\rho$. Further, the heat source $Q = \frac{Q_0}{1 - \beta t}$, is employed between the two concentric cylinders.

The mathematical formulation of the proposed model by continuity, momentum, magnetic field, energy, and mass transfer equations are as follow^26^.

Continuity equation:

1 $$\begin{aligned} \frac{1}{r} \frac{\partial }{\partial r} (r u) + \frac{\partial w}{\partial z} = 0, \end{aligned}$$

The Momentum equations with variable magnetic effect^26–28^:

2 $$\begin{aligned} \begin{aligned}{}&\frac{\partial u}{\partial t} + u \frac{\partial u}{\partial r} + w \frac{\partial u}{\partial z} = - \frac{1}{\rho } \frac{\partial P}{\partial r} + \frac{\mu }{\rho } \left( \frac{\partial ^2 u}{\partial r^2} + \frac{1}{r} \frac{\partial u}{\partial r} - \frac{u}{r^2} + \frac{\partial ^2 u}{\partial z^2}\right) - \frac{b_3 \sigma }{\rho } \left( \frac{\partial b_3}{\partial r} - \frac{\partial b_1}{\partial z}\right) \end{aligned} \end{aligned}$$

3 $$\begin{aligned} \begin{aligned}{}&\frac{\partial w}{\partial t} + u \frac{\partial w}{\partial r} + w \frac{\partial w}{\partial z} = - \frac{1}{\rho } \frac{\partial P}{\partial z} + \frac{\mu }{\rho } \left( \frac{\partial ^2 w}{\partial r^2} + \frac{1}{r} \frac{\partial w}{\partial r} + \frac{\partial ^2 w}{\partial z^2}\right) + \frac{b_1 \sigma }{\rho } \left( \frac{\partial b_3}{\partial r} - \frac{\partial b_1}{\partial z}\right) \end{aligned} \end{aligned}$$

Maxwell Equations^27,28^:

4 $$\begin{aligned}{}&\frac{\partial b_1}{\partial t} = u \frac{\partial b_3}{\partial z} + b_3 \frac{\partial u}{\partial z} - w \frac{\partial b_1}{\partial z} - b_1 \frac{\partial w}{\partial z} + \frac{1}{\sigma \mu _e} \left( \frac{\partial ^2 b_1}{\partial r^2} + \frac{1}{r} \frac{\partial b_1}{\partial r} - \frac{b_1}{r} + \frac{\partial ^2 b_1}{\partial z^2}\right) \end{aligned}$$

5 $$\begin{aligned}{}&\frac{\partial b_3}{\partial t} = - u \frac{\partial b_3}{\partial r} - b_3 \frac{\partial u}{\partial r} + w \frac{\partial b_1}{\partial r} + b_1 \frac{\partial w}{\partial r} + \frac{1}{\sigma \mu _e} \left( \frac{\partial ^2 b_3}{\partial r^2} + \frac{1}{r} \frac{\partial b_3}{\partial r} + \frac{\partial ^2 b_3}{\partial z^2}\right) \end{aligned}$$

The Energy Equation^26^:

6 $$\begin{aligned} \begin{aligned} \frac{\partial T}{\partial t} + u \frac{\partial T}{\partial r} + w \frac{\partial T}{\partial z}&= \frac{\kappa }{(\rho C_{p})} \left( \frac{\partial ^2 T}{\partial r^2} + \frac{1}{r} \frac{\partial T}{\partial r} + \frac{\partial ^2 T}{\partial z^2}\right) \\ {}&+ \frac{Q}{(\rho C_{p})} (T - T_0) \end{aligned} \end{aligned}$$

The Mass Transfer Equation^29^:

7 $$\begin{aligned} \begin{aligned} \frac{\partial C}{\partial t} + u \frac{\partial C}{\partial r} + w \frac{\partial C}{\partial z}&= D_B \left( \frac{\partial ^2 C}{\partial r^2} + \frac{1}{r} \frac{\partial C}{\partial r} + \frac{\partial ^2 C}{\partial z^2}\right) \\ {}&- k_0 (C - C_0) + \frac{D_T}{(T_m} \left( \frac{\partial ^2 T}{\partial r^2} + \frac{1}{r} \frac{\partial T}{\partial r} + \frac{\partial ^2 T}{\partial z^2}\right) \end{aligned} \end{aligned}$$

where $b_1, b_3$ are the components of magnetic field, *u* and *w* are the component of velocity field, $(\rho C_{p})$ is the heat capacity, *P* is fluid pressure, *T* is the temperature, $\rho$ is fluid density, $\sigma$ is electrical conductivity, $\mu$ is kinematic viscosity, $\kappa$ is the thermal conductivity.

### Boundary conditions

The boundary conditions of the proposed model as follow^26^:

8 $$\begin{aligned} \begin{aligned}{}&u = w = 0,b_1 = - \frac{2 M_0 \nu }{a_0 \sqrt{\xi } \sqrt{1-\beta t}}, b_3 = \frac{4 M_0 \nu z}{a^2_0 (1- \beta t)},T = T_m - T_0, C = C_{m} - C_0 at r = a(t) \\ {}&u = - \frac{2 \nu }{a_0 \sqrt{\xi } \sqrt{1-\beta t}}, w = 0, b_1 = b_3 = 0, T = C = 0, at r = b(t) \end{aligned} \end{aligned}$$

The following similarity transformations have been used for reducing a system of PDEs $(1-7)$ into a non-linear system of ODEs,

9 $$\begin{aligned} \begin{aligned}{}&u = - \frac{2 \nu }{a_0 \sqrt{\xi } \sqrt{1-\beta t}} F(\xi ), w = \frac{4 z \nu }{a^2_0 (1-\beta t)} F'(\xi ), b_{1} = - \frac{2 M_0 \nu }{a_0 \sqrt{\xi } \sqrt{1-\beta t}} G'(\xi ) , \\ {}&b_{3} = \frac{4 z M_0 \nu }{a^2_0 (1-\beta t)} G'(\xi ), \xi = \frac{r^2}{a^2_0} \frac{1}{1 - \beta t}, {\theta (\xi ) = \frac{T - T_0}{T_h - T_0}}, {\phi (\xi ) = \frac{C - C_0}{C_h - C_0}} \end{aligned} \end{aligned}$$

Therefore, Eq. (1) of the model has satisfied automatically, and the reduced form of the remaining equations $(2-7)$ are as follow:

10 $$\begin{aligned}{}&F'''' = \frac{1}{\xi } (S(2F''+\xi F''') + F'F'' - FF''' + \frac{Re_m M}{2 \xi } (SGG'+2 \xi SG'^2 - 2 F G'^2 + 2 FF' G') \nonumber \\&+ \frac{Re_m M}{\xi } (SGG' - \frac{1}{2} (F'G^2 - FGG') + F''G^2) \nonumber \\&+ \frac{Re^2_m M S}{2 \xi ^2} (\xi - F) (SG^2 + 2 \xi SGG' - 2 F G G' + 2 FF' G) \nonumber \\&+ \frac{Re_m M}{2 \xi ^2} (SG^2+2 \xi SGG' - 2 F G G' + 2 FF'G)) - \frac{2}{\xi } F''', \end{aligned}$$

11 $$\begin{aligned}{}&G'' = \frac{Re_m M}{2 \xi } (S(G+2 \xi G') - 2 F G' + 2 FF'), \end{aligned}$$

12 $$\begin{aligned}{}&\theta '' = - \frac{1}{2 \xi } \theta ' + \frac{Pr}{\xi } (S \xi - F) \theta ' + \frac{Hs}{\xi } \theta , \end{aligned}$$

13 $$\begin{aligned}{}&\phi '' = - \frac{1}{2 \xi } \phi ' + \frac{Sc}{\xi } (S \xi \phi ' - F \phi ' + C_1 S \phi + \frac{Sr Sc}{2 \xi } \theta ' - \frac{Sc Sr Pr}{\xi } (S \xi - F) \theta ' - \frac{Sc Sr Hs}{\xi } \theta , \end{aligned}$$

and the boundary conditions in the reduced form as follow,

14 $$\begin{aligned} \begin{aligned}{}&F(1) = 0, F'(1) = 0, G(1) = 1, \theta (1) = 1, \phi (1) = 1, at r = 1 \\ {}&F(k) = 1, F'(k) = 0, G(k) = 0, \theta (k) = 0, \phi (k) = 0, at r = k \end{aligned} \end{aligned}$$

where $M = \frac{ M_0^2 \sigma _f}{\rho }$ Magnetic parameter, $S = \frac{a^2_0 \alpha }{4 \nu }$ squeeze parameter, $Re_m = \sigma \nu \mu _e$ Rynold’s Magnetic parameter, $Pr = \frac{\nu (\rho Cp)}{\kappa }$ Prandtl number, $Hs = \frac{Q_0 a^2_0}{\kappa }$ heat source parameter, $Sc = \frac{\nu }{D_m}$ Schmidt number, $Sr = \frac{D_l (T_m - T_0)}{C_m - C_0}$ Soret number, $C_1 = \frac{k_0 (1-\beta t)}{\beta }$ Chemical reaction parameter, $\frac{1}{Cp} (\frac{2 \nu }{r})^2$ Eckert number and $T_0$ and $C_0$ are the ambient temperature and concentration, $T_h$ and $C_h$ are any reference temperature and concentration chosen unequal to $T_0$ and $C_0$. Further assumption, the inner cylinder are maintained at fixed temperature and concentration $T_m$ and $C_m$.

## Entropy generation

The volumetric rate in the magnetic field can be taken as a part of the entropy generation^27,28^.

15 $$\begin{aligned} \begin{aligned} N_g =&\frac{\kappa }{(T_h - T_0)^2} \left( \left( \frac{\partial T}{\partial r}\right) ^2 + \left( \frac{\partial T}{\partial z}\right) ^2\right) + \frac{\mu }{T_h - T_0} \left( 2 \left( \frac{\partial u}{\partial r}\right) ^2 + 2 (\frac{u}{r})^2 + 2 (\frac{\partial w}{\partial z})^2 + \left( \frac{\partial u}{\partial z} + \frac{\partial w}{\partial r}\right) ^2 \right) \\ {}&+ \frac{\sigma }{T_h - T_0} (u^2 b^2_3 + w^2 b^2_1 - 2 u w b_1 b_3) \end{aligned} \end{aligned}$$

the transformed form as follow as,

16 $$\begin{aligned} \begin{aligned} Ns = \frac{Ng}{Ng_{0}}&= \xi ^2 \theta '^{2} + 4 \Omega Ec Pr (F^{2} + 4 \xi ^2 F'^{2} - 2 \xi FF') \\ {}&+ 4 \Omega Ec Pr \delta (\xi ^4F''^2 + M \xi ^2 ( F^2 G'^{2} F'^{2} G^2 - 2 G F' F G' ), \end{aligned} \end{aligned}$$

where *V* is the velocity vector,$(T_{m} - T_0)$ is a reference temperature $\kappa$ is the thermal conductivity, $Ns = \frac{N_{g}}{N_{g0}}$, is the rate of entropy generation, $N_{g0} = \frac{\kappa (T_{m} - T_0)}{(T_{h} - T_0)^{2} r^{2}}$ is the entropy generation rate behavior, $\Omega = \frac{T_{h} - T_0}{T_{m} - T_0}$ is the dimensionless temperature difference. Hence, the dimensionless form of the entropy generation as follow.

17 $$\begin{aligned} Ns = N_{H} + N_{f} + N_{mf}, \end{aligned}$$

Where $N_{f}$, $N_{H}$ and $N_{mf}$ are denoting the entropy generation due friction of the fluid, entropy generation due to heat transfer and local entropy generation due to magnetic field respectively. The viscosity and heat transfer effects are described in the Bejan number as follows.

18 $$\begin{aligned} Be = \frac{N_{H}}{Ns} = \frac{\xi ^2 \theta '^{2} (1-R_{1} \theta )}{Ns}. \end{aligned}$$

Emerging physical parameters in the reduced form of system are the Nusselt number and skin friction coefficient, and can be defined as,

19 $$\begin{aligned} C_{f} = \frac{1}{\nu r Re} \left( \frac{\partial w}{\partial r} \right) _{r=b(t)}, \ \ \ \ N_{u} = - \frac{r \kappa \left( \frac{\partial T}{\partial r} \right) _{r=b(t)}}{k (T_m - T_0)}, \end{aligned}$$

In case of Eq. (19), we get

20 $$\begin{aligned} \frac{Re^{2}}{8z} C_{f} = f''(2), \ \ \ \ - \theta '(2) = \frac{N_{u}}{2}. \end{aligned}$$

## Numerical solution by PCM

In this section, optimal choices of continuation parameters are made through the algorithm of PCM^27^ for the solution of non-linear equations $(10-13)$ with boundary conditions in equation (14):

- **First order of ODE** To transform the equations $(10-13)$ into first order of ODE’s, consider the following
  21 $$\begin{aligned} \begin{aligned}{}&F = s_{1}, F' = s_{2}, F'' = s_{3}, F''' = s_{4} \\ {}&G = s_{5}, G' = s_{6}, \theta = s_{7}, \theta ' = s_{8}, \phi = s_{9}, \phi ' = s_{10} \end{aligned} \end{aligned}$$
  putting these transformations in Eqs. $(10-13)$, which becomes
  22 $$\begin{aligned}{}&s'_{4} = \frac{1}{\xi } (S(2s_{3}+\xi s_{4}) + s_{2} s_{3} - s_{1} s_{4} + \frac{Re_m M}{2 \xi } (S s_{5} s_{6}+2 \xi S s_{6}^2 - 2 s_{1} s_{6}^2 + 2 s_{1} s_{2} s_{6}) \nonumber \\&+ \frac{Re_m M}{\xi } (S s_{5} s_{6} - \frac{1}{2} (s_{2} s_{5}^2 - s_{1} s_{5} s_{6}) + s_{3} s_{5}^2) \nonumber \\&+ \frac{Re^2_m M S}{2 \xi ^2} (\xi - s_{1}) (Ss_{5}^2 + 2 \xi S s_{5} s_{6} - 2 s_{1} s_{5} s_{6} + 2 s_{1} s_{2} s_{5}) \nonumber \\&+ \frac{Re_m M}{2 \xi ^2} (S s_{5}^2+2 \xi S s_{5} s_{6} - 2 s_{1} s_{5} s_{6} + 2 s_{1} s_{2} s_{5})) - \frac{2}{\xi } s_{4}, \end{aligned}$$
  23 $$\begin{aligned}{}&s_{6}' = \frac{Re_m M}{2 \xi } (S(s_{5}+2 \xi s_{6}) - 2 s_{1} s_{6} + 2 s_{1} s_{2}), \end{aligned}$$
  24 $$\begin{aligned}{}&s_{8}' = - \frac{1}{2 \xi } s_{8} + \frac{Pr}{\xi } (S \xi - s_{1}) s_{8} + \frac{Hs}{\xi } s_{7}, \end{aligned}$$
  25 $$\begin{aligned}{}&s_{10}' = - \frac{1}{2 \xi } \phi ' + \frac{Sc}{\xi } (S \xi s_{10} - s_{1} s_{10} + C_1 S s_{9} + \frac{Sr Sc}{2 \xi } s_{8} - \frac{Sc Sr Pr}{\xi } (S \xi - s_{1}) s_{8} - \frac{Sc Sr Hs}{\xi } s_{7}, \end{aligned}$$
  and the boundary conditions becomes
  26 $$\begin{aligned} \begin{aligned}{}&s_{1}(1) = 0, s_{2}(1) = 0, s_{5}(1) = 1, s_{7}(1) = 1, s_{9}(1) = 1, at r = 1 \\ {}&s_{1}(k) = 1, s_{2}(k) = 0, s_{5}(k) = 0, s_{7}(k) = 0, s_{9}(k) = 0, at r = k \end{aligned} \end{aligned}$$
- **Introducing of parameter p and we obtained ODEs in a p-parameter group** To get ODE’s in a p-parameter group, let we know p-parameter in Eqs. $(22-25)$ and therefore,
  27 $$\begin{aligned} s'_{4}&= \frac{1}{\xi } (S(2s_{3}+\xi s_{4}) + s_{2} s_{3} - s_{1} (s_{4} - 1) q + \frac{Re_m M}{2 \xi } (S s_{5} s_{6}+2 \xi S s_{6}^2 - 2 s_{1} s_{6}^2 + 2 s_{1} s_{2} s_{6}) \nonumber \\&+ \frac{Re_m M}{\xi } (S s_{5} s_{6} - \frac{1}{2} (s_{2} s_{5}^2 - s_{1} s_{5} s_{6}) + s_{3} s_{5}^2) \nonumber \\&+ \frac{Re^2_m M S}{2 \xi ^2} (\xi - s_{1}) (Ss_{5}^2 + 2 \xi S s_{5} s_{6} - 2 s_{1} s_{5} s_{6} + 2 s_{1} s_{2} s_{5}) \nonumber \\ {}&+ \frac{Re_m M}{2 \xi ^2} (S s_{5}^2+2 \xi S s_{5} s_{6} - 2 s_{1} s_{5} s_{6} + 2 s_{1} s_{2} s_{5})) - \frac{2}{\xi } s_{4}, \end{aligned}$$
  28 $$\begin{aligned}{}&s_{6}' = \frac{Re_m M}{2 \xi } (S(s_{5}+2 \xi s_{6}) - 2 s_{1} (s_{6} - 1) q + 2 s_{1} s_{2}), \end{aligned}$$
  29 $$\begin{aligned} s_{8}'&= - \frac{1}{2 \xi } s_{8} + \frac{Pr}{\xi } (S \xi - s_{1}) (s_{8} - 1) q + \frac{Hs}{\xi } s_{7}, \end{aligned}$$
  30 $$\begin{aligned} s_{10}'&= - \frac{1}{2 \xi } \phi ' + \frac{Sc}{\xi } (S \xi s_{10} - s_{1} (s_{10} - 1) q + C_1 S s_{9} + \frac{Sr Sc}{2 \xi } s_{8} - \frac{Sc Sr Pr}{\xi } (S \xi - s_{1}) s_{8} - \frac{Sc Sr Hs}{\xi } s_{7}, \end{aligned}$$
- **Differentiation by p, reaches at the following system w.r.t the sensitivities to the parameter-p** Differentiating the Eqs. $(27-30)$ w.r.t by *p*
  31 $$\begin{aligned} d'_{1} = h_{1} d_{1} + e_{1} \end{aligned}$$
  where $h_{1}$ is the coefficient matrix, $e_{1}$ is the remainder and $d_{1} = \frac{dp_{i}}{d\tau }$, $1\le i \le 10$.
- **Cauchy Problem**
  32 $$\begin{aligned} d_{1} = y_{1} + a1 v_1, \end{aligned}$$
  where $y_{1}$, $v_1$ are vector functions. By resolving the two Cauchy problems for every component. We are satisfied then automatically to ODE’s
  33 $$\begin{aligned} e_{1} + h_{1}(a1 v_1 + y_{1}) = (a1 v_1 + y_{1})^{'} \end{aligned}$$
  and left the boundary conditions.
- **Using by Numerical Solution** An absolute scheme has been used for the resolution of the problem
  34 $$\begin{aligned}{}&\frac{v_1^{i + 1} - v_1^{i}}{\triangle \eta } = h_{1} v_1^{i + 1} \end{aligned}$$
  35 $$\begin{aligned}{}&\frac{y^{i + 1} - y^{i}}{\triangle \eta } = h_{1} y^{i + 1} + e_{1} \end{aligned}$$
- **Taking of the corresponding coefficients** As given boundaries are usually applied for $p_{i}$, where $1 \le i \ \le 10$, for the solution of ODE’s, we required to apply $d_{2} = 0$, which seems to be in matrix form as
  36 $$\begin{aligned} l_{1} . d_{1} = 0 \text {or} l_{1} . (a1 v_1 + y_{1}) = 0 \end{aligned}$$
  where $$a1 = \frac{-l_{1} . y_{1}}{l_{1} . v_1}$$

**Table 1:** Comparison of the numerical results by two methods PCM and BVP4C for Skin friction and Nusselt number, with various physical parameters.

*S*.	PCM	BVP4C	PCM	BVP4C
	$f''(1)$.	$f''(1)$.	$-\theta '(1)$.	$-\theta '(1)$.
0.0	−4.5583	−4.5583	0.4785	0.4785
0.2	−4.7652	−4.7652	0.6137	0.6137
0.4	−4.9765	−4.9765	0.7754	0.7754
0.6	−5.1924	−5.1924	0.9650	0.9650
0.8	−5.4129	−5.4129	1.1829	1.1829
1.0	−5.6376	−5.6376	1.4286	1.4286
1.2	−5.8666	−5.8666	1.7007	1.7007
1.4	−6.0997	−6.0997	1.9973	1.9973
1.6	−6.3367	−6.3367	2.3158	2.3132

**Table 2:** Comparison of the numerical results by two methods PCM and BVP4C for Skin friction and Nusselt number, with various physical parameters.

*Ha*.	PCM	BVP4C	PCM	BVP4C
	$f''(1)$.	$f''(1)$.	$-\theta '(1)$.	$-\theta '(1)$.
0.0	−6.0586	−6.0586	2.3946	2.3946
0.2	−6.0811	−6.0811	2.3952	2.3952
0.3	−6.0924	−6.0924	2.3955	2.3955
0.6	−6.1263	−6.1263	2.3963	2.3963
0.8	−6.1490	−6.1490	2.3968	2.3968
0.9	−6.1604	−6.1604	2.3971	2.3971
1.2	−6.1947	−6.1947	2.3980	2.3980
1.5	−6.2292	−6.2292	2.3988	2.3988
1.8	−6.2639	−6.2639	2.3997	2.3997

**Table 3:** Comparison of the numerical results by two methods PCM and BVP4C for Skin friction and Nusselt number, with various physical parameters.

*Pr*.	PCM	BVP4C	PCM	BVP4C
	$f''(1)$.	$f''(1)$.	$-\theta '(1)$.	$-\theta '(1)$.
0.1	−5.0839	−5.0839	0.7529	0.7529
0.5	−5.0839	−5.0839	0.7744	0.7744
1.0	−5.0839	−5.0839	0.8013	0.8013
1.5	−5.0839	−5.0839	0.8285	0.8285
2.0	−5.0839	−5.0839	0.8557	0.8557
2.5	−5.0839	−5.0839	0.8831	0.8831
3.0	−5.0839	−5.0839	0.9106	0.9106
3.5	−5.0839	−5.0839	0.9381	0.9381
4.0	−5.0839	−5.0839	0.9657	0.9657

## Results and discussions

**Figure 2:**
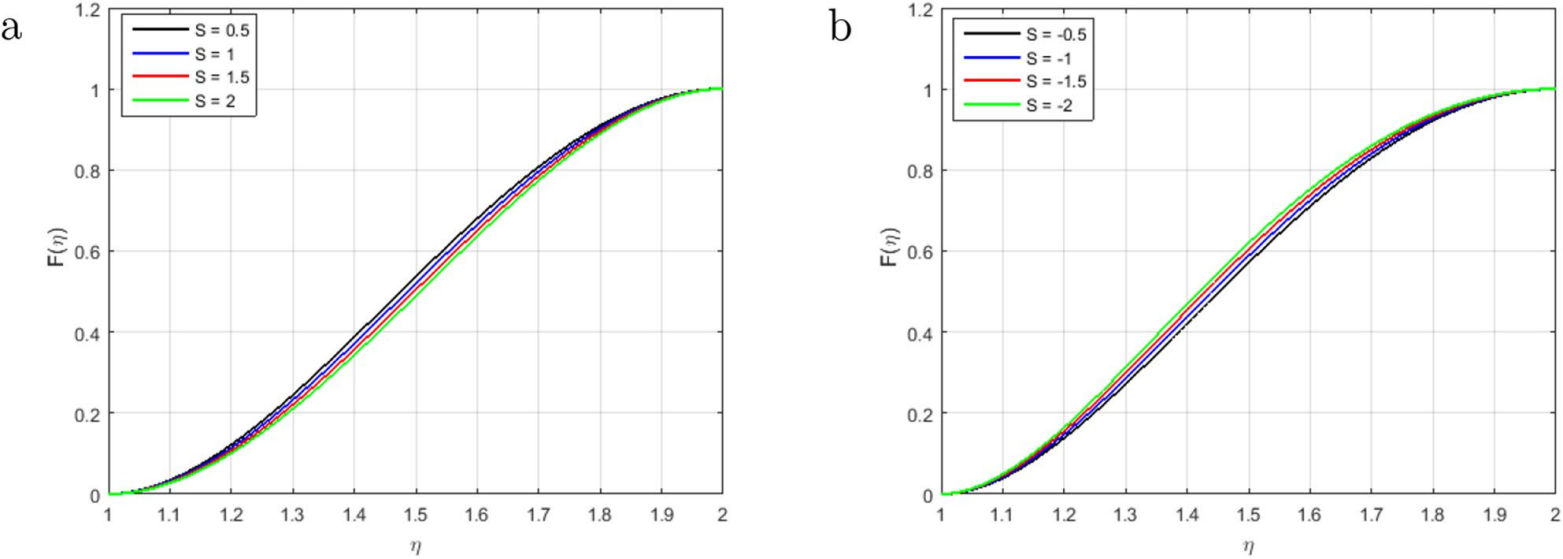
Influence of $F(\eta )$ for $S > 0$, $S < 0$ and fixed values of $Re_m = 2.5, M = 0.5, Pr = 3.2, Hs = 0.2, C1 = 0.4, Sc = 0.4, Sr = 0.5$.

**Figure 3:**
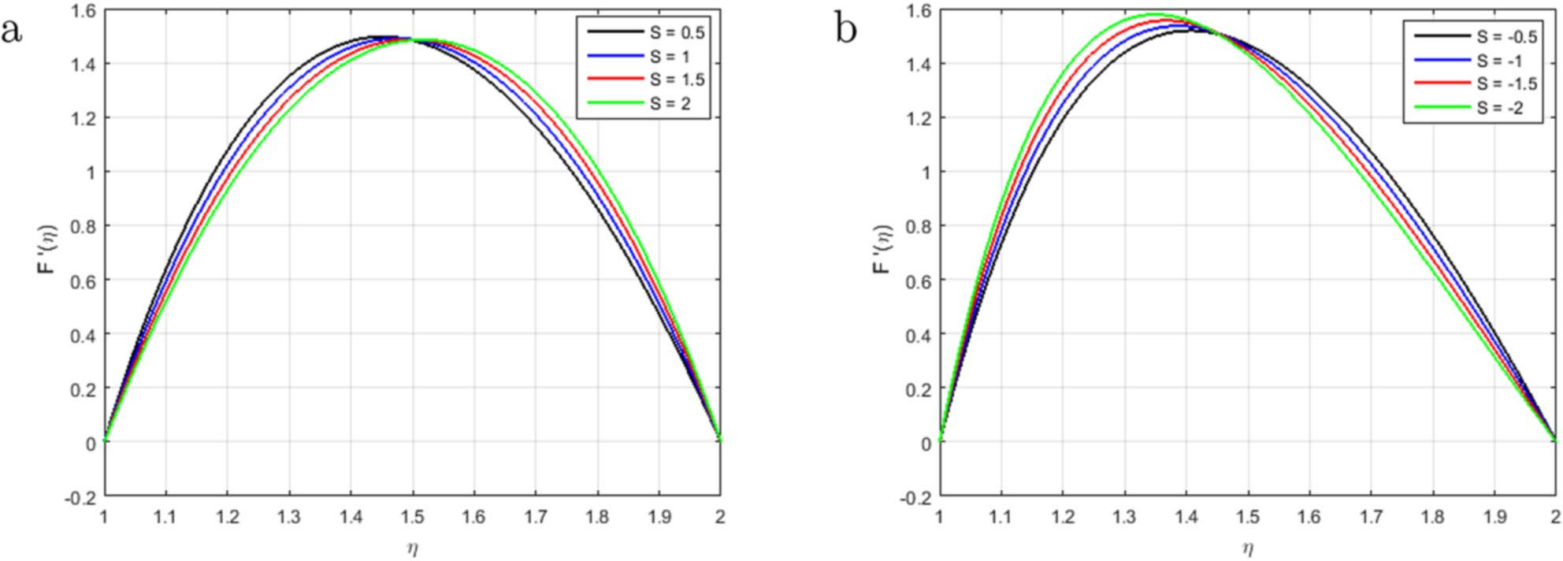
Influence of $F'(\eta )$ for $S > 0$, $S < 0$ and fixed values of $Re_m = 2.5, M = 0.5, Pr = 3.2, Hs = 0.2, C1 = 0.4, Sc = 0.4, Sr = 0.5$.

**Figure 4:**
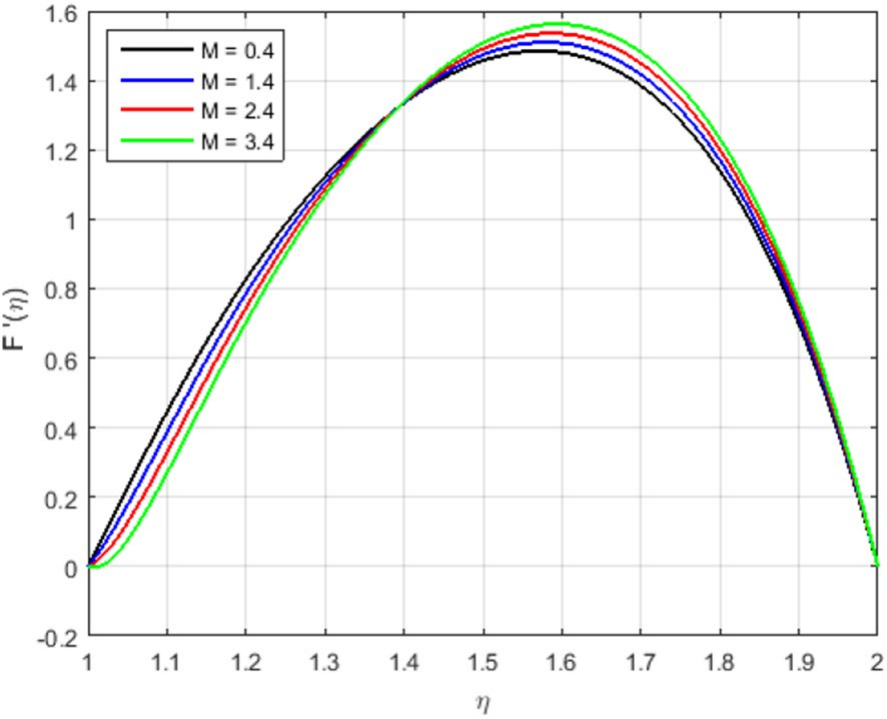
Influence of $F'(\eta )$ for *M* and fixed values of $S = 2.5, Pr = 6.2, Ec = 0.5, \delta = 0.4, \Phi _1 = 0.02, \Phi _2 = 0.5, m = 0.3$.

**Figure 5:**
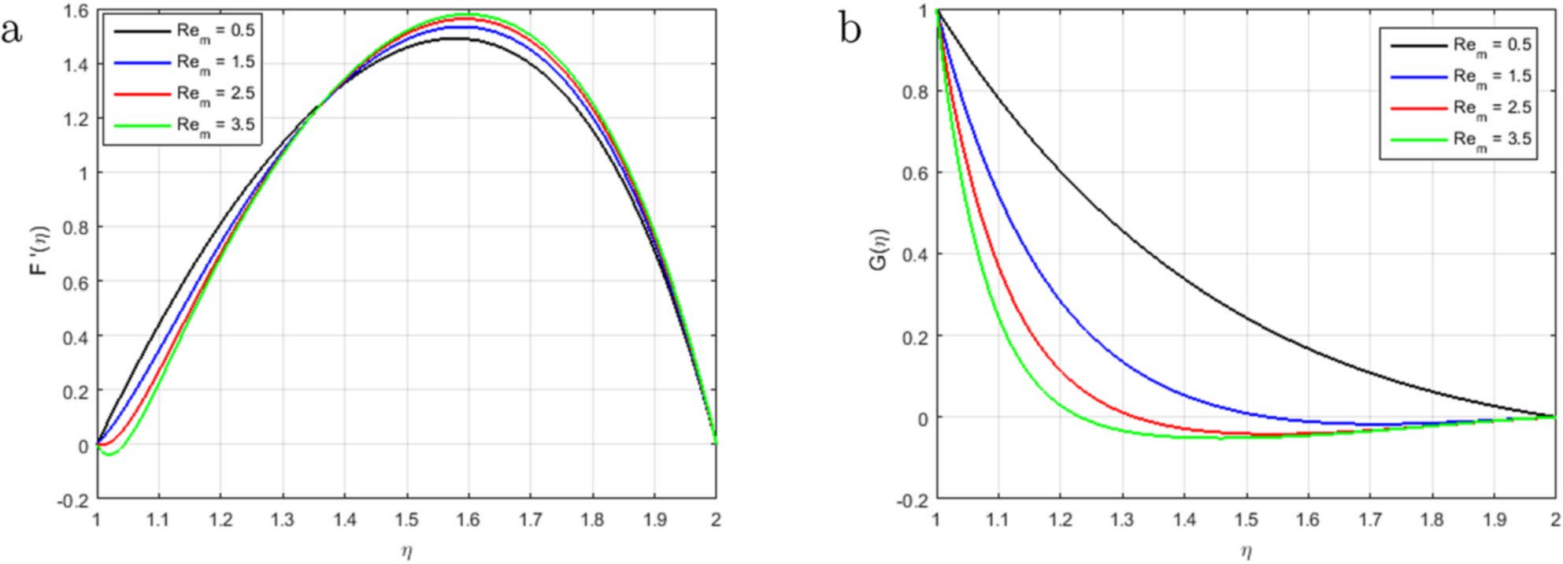
Influence of $F'(\eta )$ and $G(\eta )$ for $Re_m$ and fixed values of $S = 3.5, M = 3.4, Pr = 6.2, Hs = 1.2, C1 = 1.4, Sc = 1.4, Sr = 1.5$.

**Figure 6:**
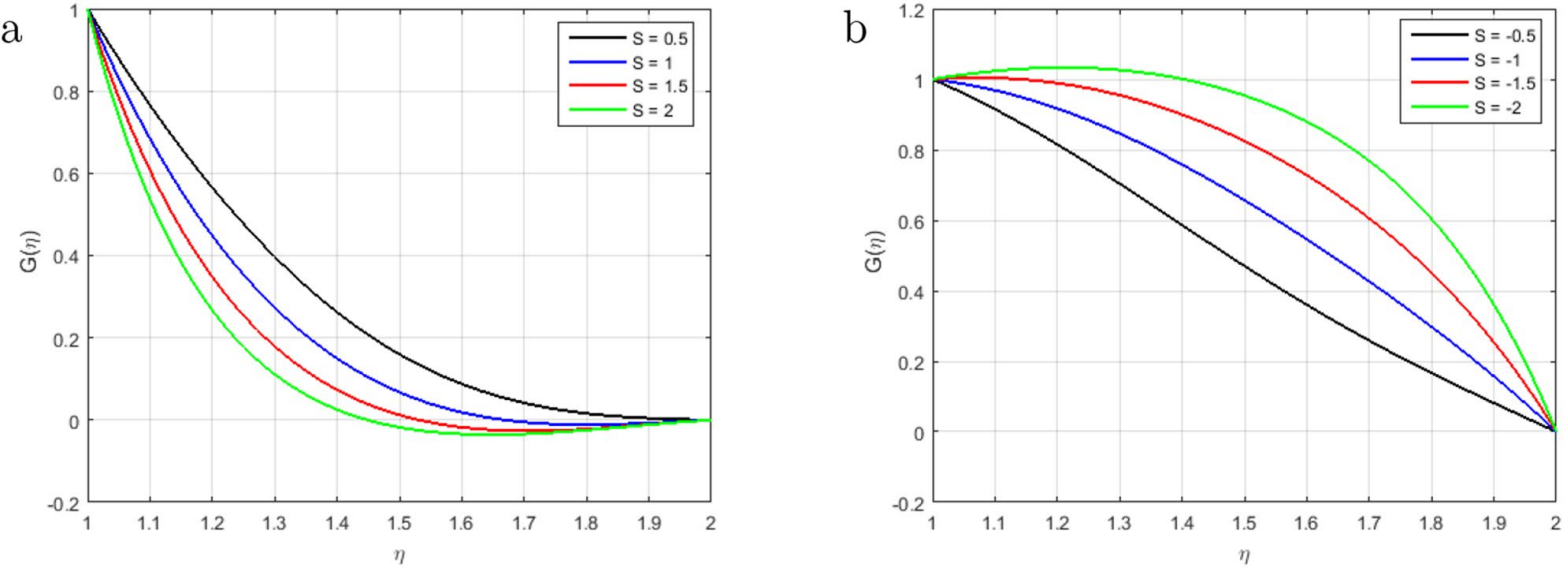
Influence of $G(\eta )$ for $S > 0$, $S < 0$ and fixed values of $Re_m = 2.5, M = 0.5, Pr = 3.2, Hs = 1.2, C1 = 0.4, Sc = 1.4, Sr = 1.5$.

**Figure 7:**
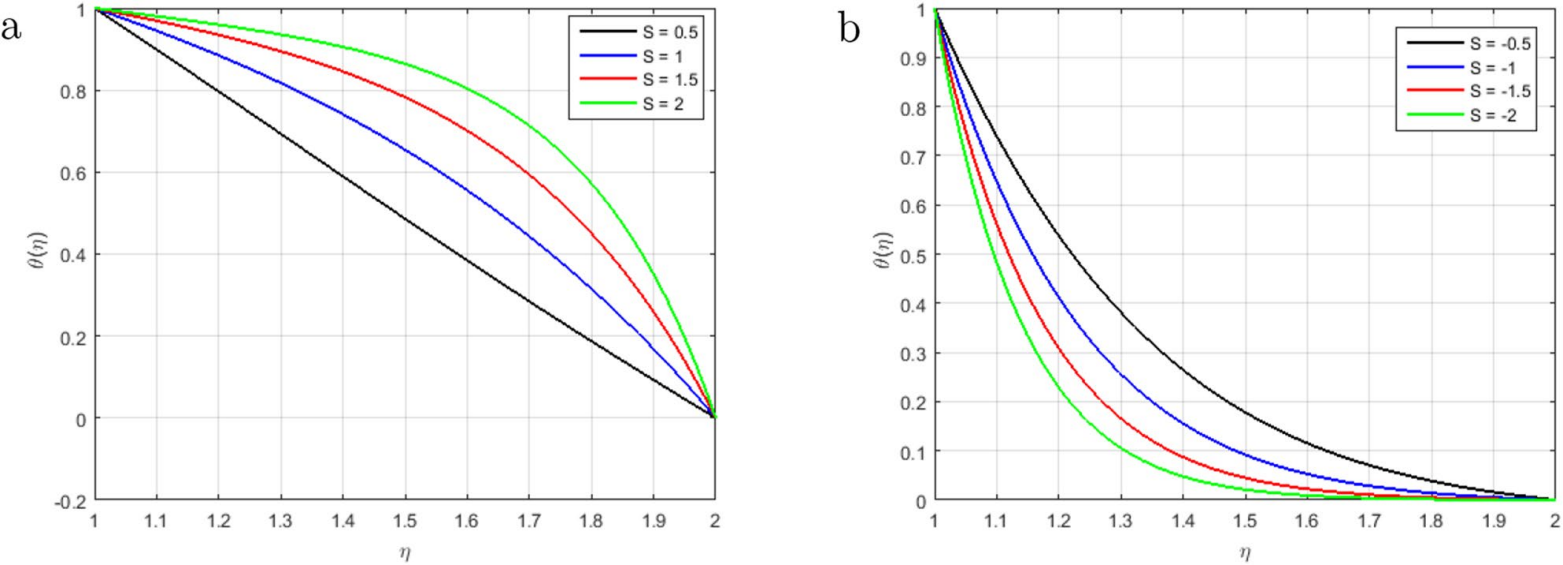
Influence of $\theta (\eta )$ for $S > 0$, $S < 0$ and fixed values of $Re_m = 2.5, M = 0.5, Pr = 3.2, Hs = 1.2, C1 = 0.4, Sc = 1.4, Sr = 1.5$.

**Figure 8:**
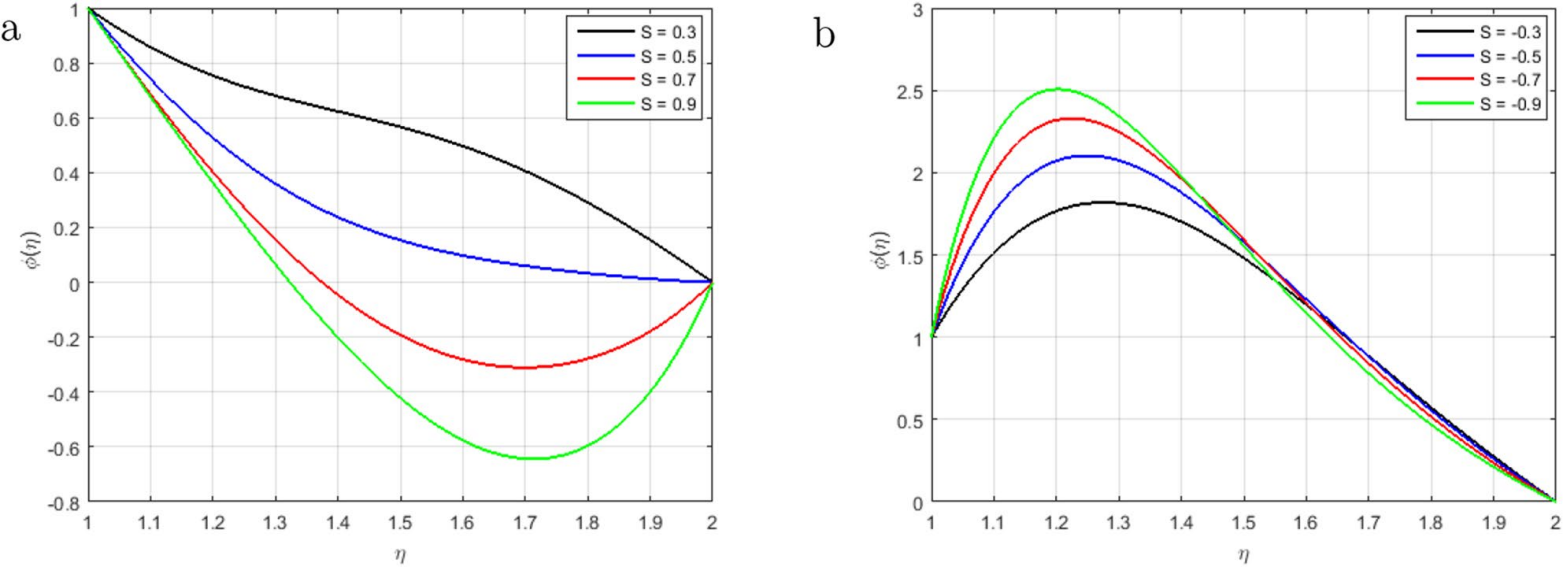
Influence of $\phi (\eta )$ for $S > 0$, $S < 0$ and fixed values of $Re_m = 2.5, M = 0.5, Pr = 6.2, Hs = 1.2, C1 = 1.4, Sc = 1.4, Sr = 2.5$.

**Figure 9:**
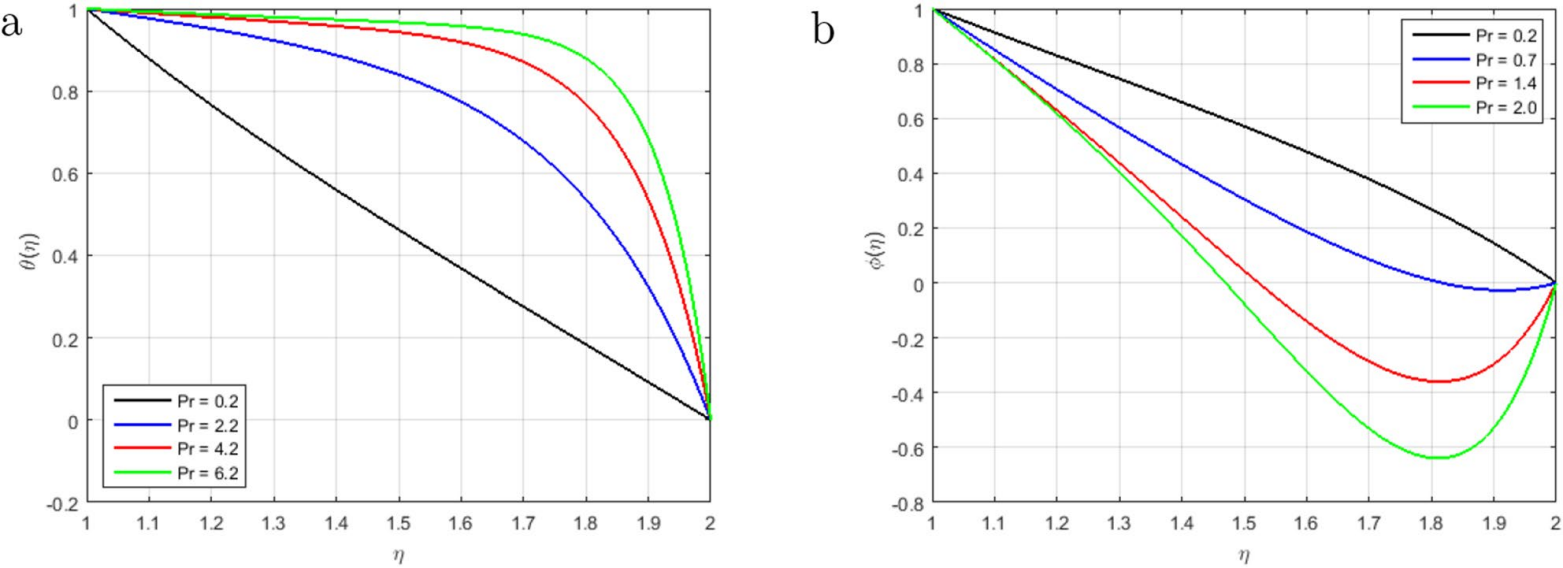
Influence of $\theta (\eta )$ and $\phi (\eta )$ for *Pr* and fixed values of $S = 2.5, M = 1.4, Re_m = 2.5, Hs = 1.2, C1 = 1.4, Sc = 1.4, Sr = 2.5$.

**Figure 10:**
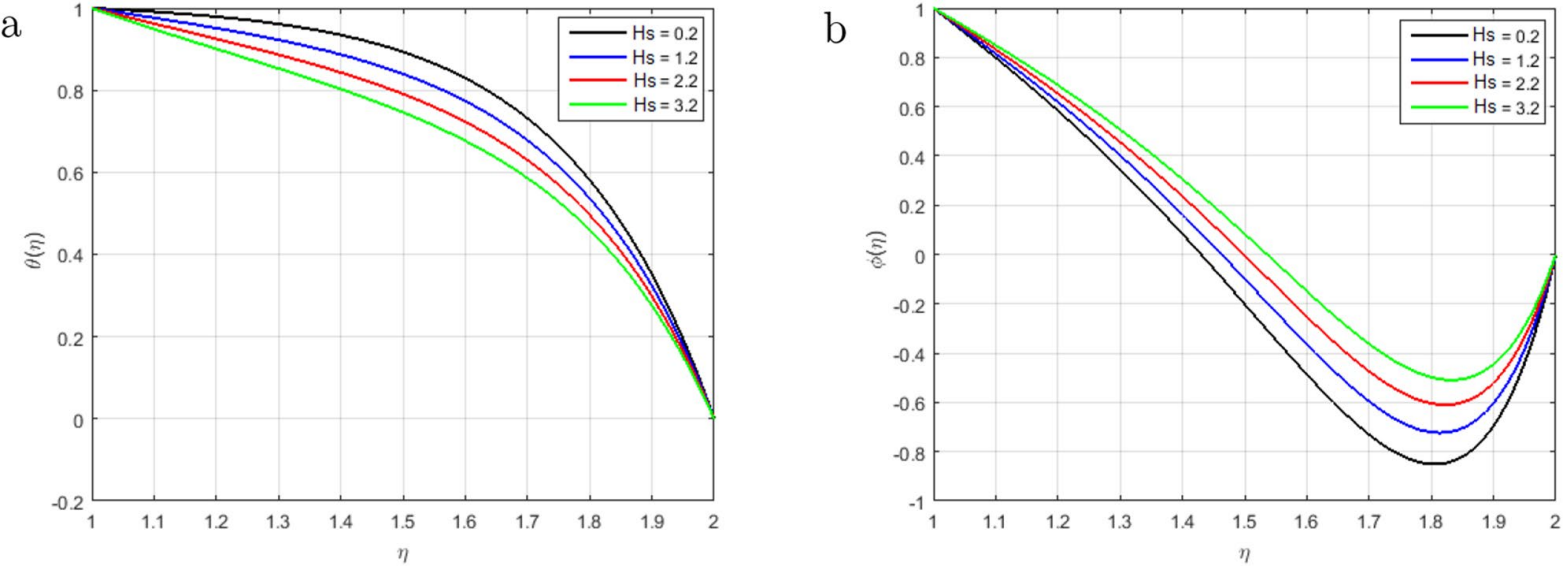
Influence of $\theta (\eta )$ and $\phi (\eta )$ for *Hs* and fixed values of $S = 2.5, M = 1.4, Pr = 2.2, Re_m = 2.5, C1 = 1.4, Sc = 1.4, Sr = 2.5$.

**Figure 11:**
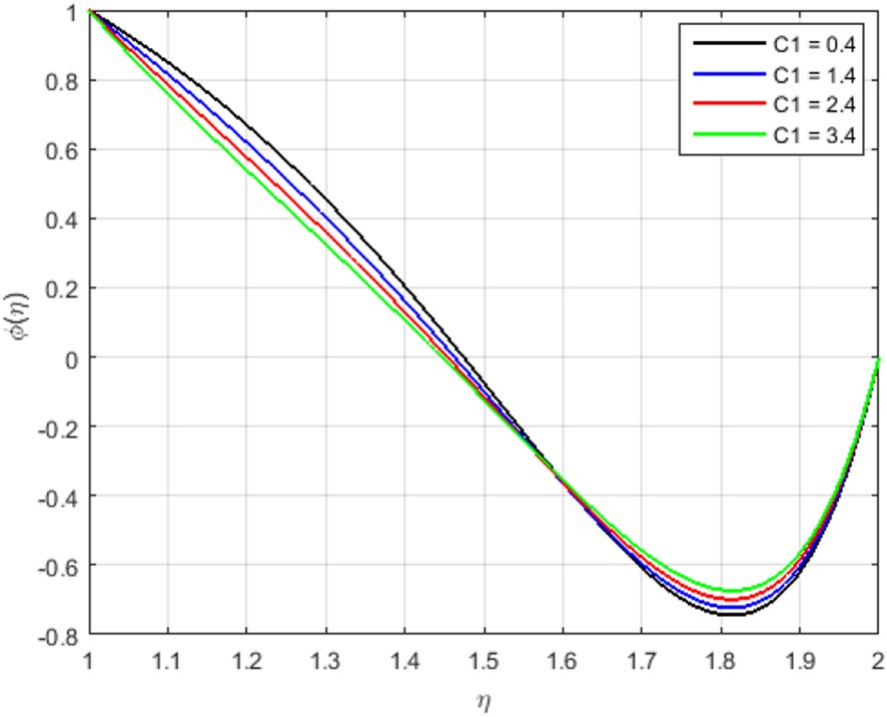
Influence of $\phi (\eta )$ for *C*1 and fixed values of $S = 2.5, M = 1.4, Pr = 2.2, Re_m = 2.5, Hs = 1.2, Sc = 1.4, Sr = 2.5$.

**Figure 12:**
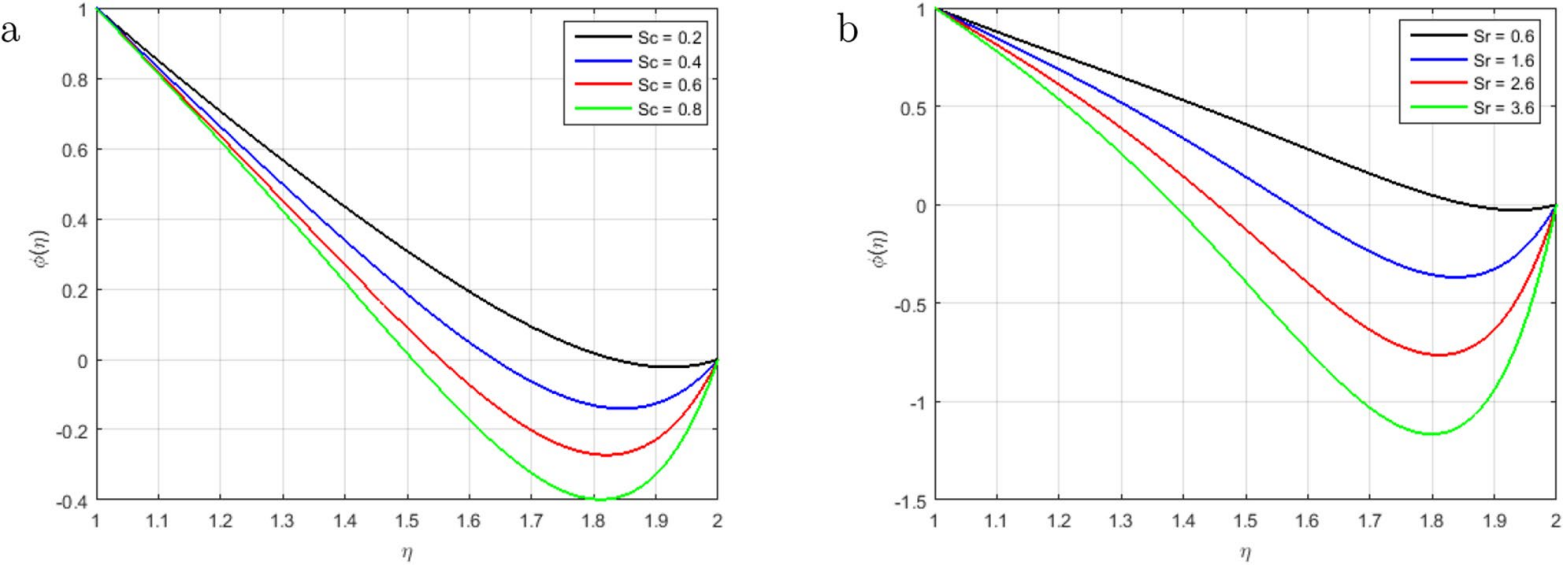
Influence of $\phi (\eta )$ for *Sc* and *Sr* and fixed values of $S = 2.5, M = 1.4, Pr = 2.2, Re_m = 2.5, C1 = 1.4, Hs = 1.2$.

**Figure 13:**
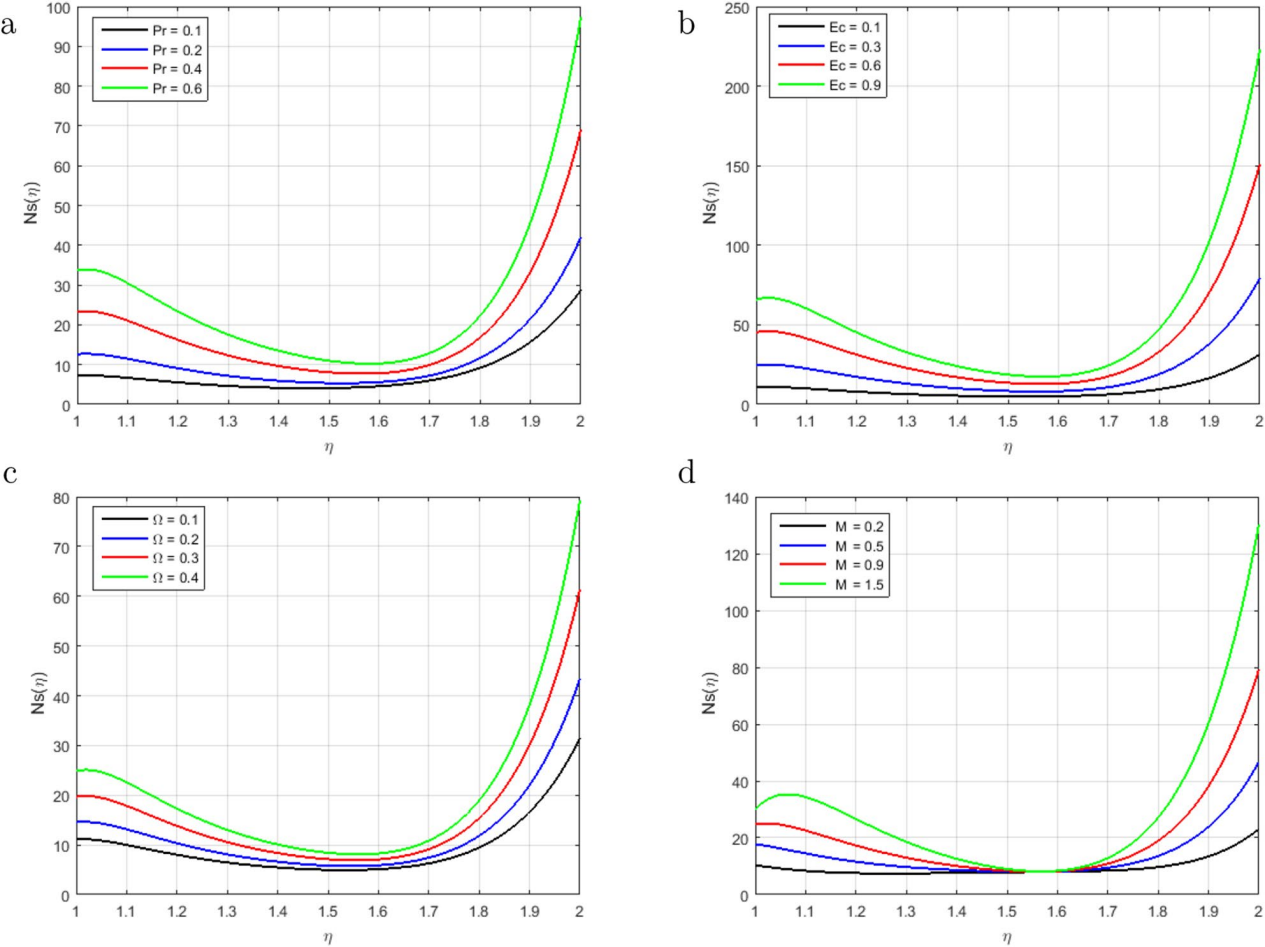
Influence of $Ns(\eta )$ for *Pr*, *Ec*, $\Omega$ and *M* and fixed values of $S = 3.3, Re_m = 1.7, C1 = 1.6, Hs = 2.2, Sc = 0.9, Sr = 1.6, \delta = 0.5$.

**Figure 14:**
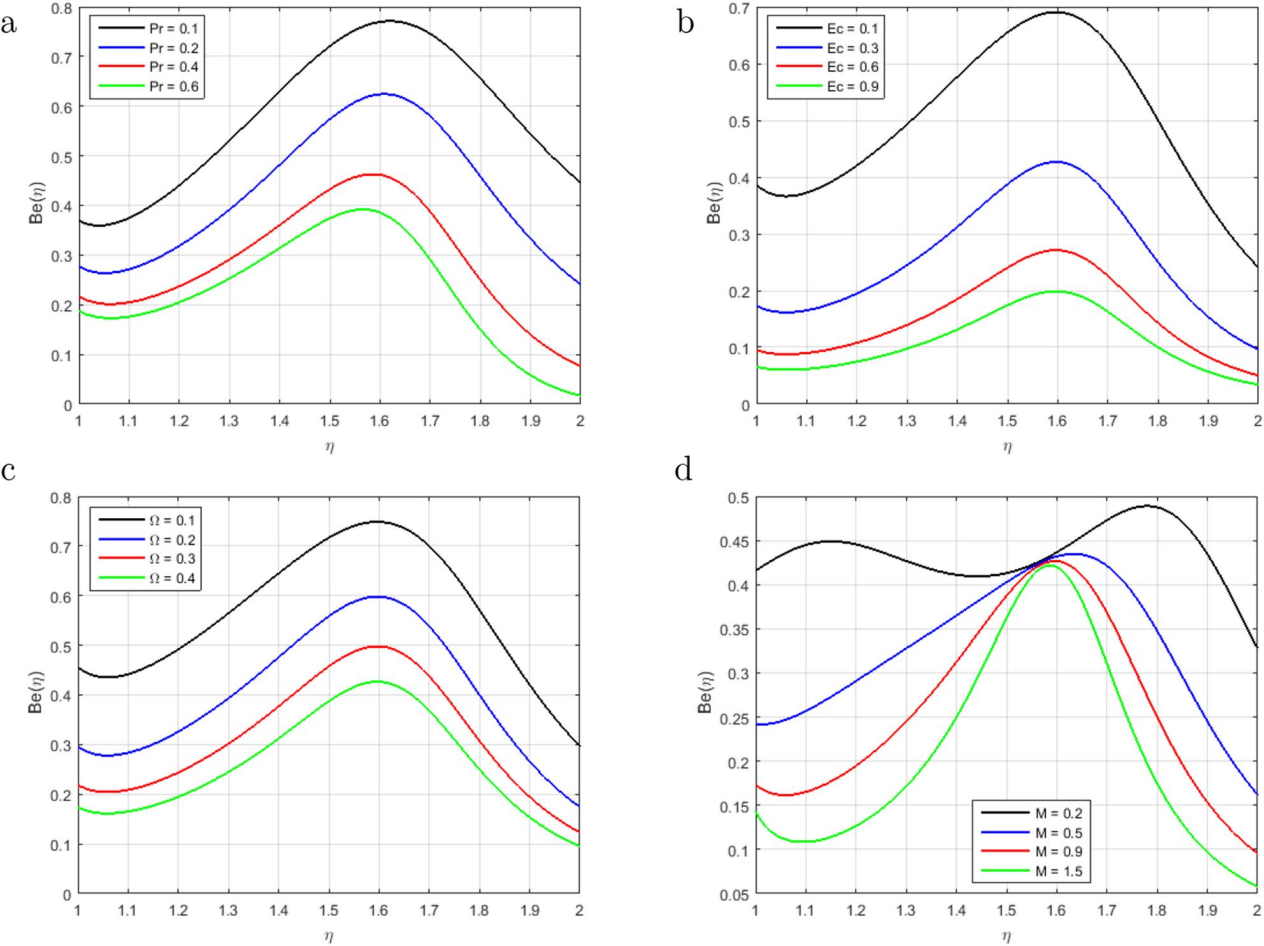
Influence of $Be(\eta )$ for *Pr*, *Ec*, $\Omega$ and *M* and fixed values of $S = 3.3, Re_m = 1.7, C1 = 1.6, Hs = 2.2, Sc = 0.9, Sr = 1.6, \delta = 0.5$.

We have analyzed time-dependent non-compressible two-dimensional squeezing flow between concentric cylinders concerning heat transfer and the behavior of the magnetic effect. The impacts of various emerging parameters, including Prandtl number *Pr*,Magnetic number *M*, squeezing parameter *S*, Schmidt parameter *Sc*, magnetic Reynolds number $Re_m$, Soret parameter *Sr*, Chemical reaction parameter *C*1, Eckert number *Ec* and difference temperature $\Omega$ have been investigated regarding the fluid flow, heat transmission, mass transfer, entropy generation and Bejan number in Figs. 2, 3, 4, 5, 6, 7, 8, 9, 10, 11, 12, 13 and 14. It is noteworthy that the outcomes of our proposed model produce better effects than the current model in the literature. Tables 1, 2, 3) are described the numerical results of two important flow parameters, skin friction and Nusselt number, which are acquired in MATLAB by two different numerical schemes (BVP4C and PCM).

It is indispensable to record that the squeezing number *S* explains the flow in the concentric cylinder as shown in Fig. 2a, where $S > 0$represents the two cylinders moving apart and $S < 0$represents the opposite trend of the cylinder for the so-called squeezing flow. In this ongoing analysis, the positive values of $S >0$ are taken. As long as the value of the squeezing number *S* is incrementing, the horizontal velocity components decrement gradually. It is also necessary to mention that the influence of the magnetic field is declining the intensity of the velocity throughout the domain under consideration which gives existence to a new force known as Lorentz force. This force acts in the opposite direction of the flow when the magnetic field is employed in the orthogonal direction to the flow. Such kind of opposing force gets slows the velocity of the fluid. Figure 2b illustrates the impact of squeezing number *S* at the horizontal components of the fluid velocity $f(\eta )$. Therefore, it is also noteworthy that incrementing the negative value of the squeezing number *S* expands the flow channel thus decreasing the horizontal components of the velocity $f(\eta )$ with the increase in the absolute value of $S < 0$. Figure 3a,b describe the behavior of the squeezing parameter *S*concerning two parallel concentric cylinders, the channel of the flow between two parallel concentric cylinders expands when $S >0$, as a result, the vertical components of the velocity $f'(\eta )$ decrements in the domain $\eta < 1.5$, and increments in the domain $\eta > 1.5$. Similarly, the channel of the flow between two parallel concentric cylinders contracts when $S <0$, as a result, the vertical components of the velocity $f'(\eta )$ increments in the domain $\eta < 1.45$, and decrements onwards. Figure 4 illustrates the impact of magnetic parameter *M*, while Fig. 5a explains the effect of magnetic Reynold’s number $Re_m$concerning horizontal velocity in the flow direction. It can be seen in Figures 4 and 5a that the horizontal components of fluid velocity decrements with the increase in the Magnetic parameter *M* and magnetic Reynold’s number $Re_m$. The fact that intensifying the magnetic field causes the opposite force to the flow to rise is known as the Lorentz force. This force acts opposite to the flow direction, and hence, the velocity gets decreases but it is completely showing opposite behavior for the flow domain, where $\eta > 1.4$. The velocity profile is maximum in the center of the annulus cylinder, and the velocity profile follows a parabolic type shape vertically in the upward direction. Figure 5b illustrates the effect of the radius of the inner cylinder and the magnetic Reynolds number $Re_m$ on the magnetic profile, which shows that the electron is moving from outer to inner; in a conventional sense, we can say the current is flowing from inner to outer. On the other hand, If the $Re_m$, rises gradually, the fluid’s velocity approaches the boundary and is negligible far away from it. This is obvious that the profile of the magnetic field decrements with increase in $Re_m$.

Figure 6a illustrates that, as long as the value of the squeezing parameter *S* increments, the magnetic field’s profile decrements due to the existence of Lorentz force, which gets slows the motion of the fluid. In addition, when $S<0$, the annulus formed by an outer and inner cylinder contracts, and two of the cylinders get close to one another, this condition, together with declining Lorentz force, produces an unfavorable pressure gradient. Furthermore, the magnetic profile increments with the increase in the intensity of the squeezing parameter $S > 0$, as shown in Fig. 6b. It has been noticed from the graph that the magnetic field can be utilized to improve the fluid flow; as a result, the squeezing number increases, which augment the magnetic field. Figure 7a explains the impact of $S > 0$ on $\theta (\eta )$ profile. For the higher positive values of *S*, the temperature profile rises, as the kinetic energy of the molecules rises and the attraction between molecules weakens. Therefore, the viscosity of a fluid decrements with an increment temperature profile. The effect of squeezing parameter $S < 0$ on temperature in Fig. 7b. It is shown that due to the increase in the attractive binding energy and hence, the temperature profile reduces. In Fig. 8a, it is displayed that the concentration profile decrements with the augmenting value of the squeezing number $S > 0$, and thus, more squeezing generates cooling effects in the viscous liquid system. Figure 8b depicts the impact of squeezing number *S* on the concentration profile, and it shows that the concentration profile augments with the rise in the squeezing number *S* when $\eta < 1.5$. Nevertheless, it decrements with the increments of these parameters when $\eta > 1.5$. Figure 9a,b shown that the increment in Prandtl number *Pr* increases heat transfer and decreases mass transfer due to the increments the kinetic energy that move faster i.e.. They are running their bearing charge fast. As a result, the conductivity rises. Hence, the temperature decreases with increasing Pr. An increase in the Prandtl number rises the thermal boundary layer thickness. The ratio of momentum diffusivity to thermal diffusivity is referred to Prandtl number.

Therefore, an increment in the Prandtl number indicates that the momentum spreads rapidly and that the velocity boundary layer is relatively thicker than the layer of the thermal boundary, resulting in less heat transfer through bulk motion. This means that the convection dominates the conduction. Therefore, the mass transfer decrements with an increment in *Pr*. The impact of heat source *Hs* on concentration and temperature profiles is shown in Fig. 10a,b. The temperature profile decrements and the mass transfer increments and at the same time becomes linear at the augmenting value of the heat source *Hs*. Due to the domination of the heat absorption coefficient, we have observed a raised in the velocity profiles of the flow. Figure 11 explains the impact of the chemical reaction parameter *C*1 on the concentration profile. We can observe from the graph that the concentration profile is greatly influenced by the values of the chemical reaction parameter cause an increase in chemical molecule diffusivity and hence retards in the flow domain. However, the behavior of the flow becomes opposite in the region where $\eta > 1.6$. Figure 12a illustrates the impact of Schmidt number *Sc*, and it has been noticed that the increment in Schmidt number *Sc* decrements the mass transfer due to the thermal boundary layer thickness decreases. The reality is that the larger values of *Sc* have smaller diffusivity of mass, which causes a thinner concentration boundary layer. As the molecular diffusion rate is dominated the viscous diffusion rate, the concentration profile decreases. Figure 12b explains the impact of the Soret parameter *Sr* at the concentration profile. In this paper, the following values of *Sr* are taken as *Sr* = 0.1, 0.4, 0.7, 1.0, it shows that the values of the Soret parameter are augmented. The contribution of concentration gradients to the thermal energy flux in a fluid flow is shown by the Soret number *Sr*. A rise in the Soret number *Sr* is associated with an increase in velocity and temperature, as well as a decrease in concentration.

Figure 13a–c illustrate the behavior of entropy generation, it has been observed that entropy generation augments in the entire domain with the increment in *Ec*, *Pr*, and $\Omega$. Whereas minor variation is seen in the middle of the cylinder. The impacts of entropy generation with the rising value of the *Pr* are sketched in Fig. 13a. It has been observed that entropy generation is optimized for a fixed value at the stretching surface inside the domain and slows down as the distance from the sheet increases. Further, the surface of the stretching sheet is a good source of entropy generation because heat transfer and fluid friction are significant at the surface. So, as shown in figure, the entropy generation increases due to the viscous dissipation thus encourages heat irreversibility because of heat transport within the flow channel. The *Ec* is sketched in Fig. 13b describes the thermal energy heating measurement, it shows that the Eckert number is producing more entropy generation under the influence of stretching surface. As a result, decreases the Eckert number achieves the basic goal of the second law of thermodynamics, which is to minimize the entropy genera rate per unit volume. The influence of increasing *Ec* on entropy generation is most noticeable near the stretched sheet’s surface, but it has no significant influence in the main flow domain. The effect of $\Omega$ on entropy generation in Fig. 13c, consequently increasing the molecular disorganization and, therefore, Increasing the entropy production within the boundary layer. Moreover, heat is a disorganized shape of energy that increases due to the increment in $\Omega$ of viscous heat, so entropy construction increases. Therefore, entropy construction will be minimal due to low viscous heating. No considerable impact of the magnetic parameter *M* at the dimensionless form of total entropy generation is noticed in the middle of the channel as shown in Fig. 13d. There is a consistency in the various curves of the entropy generation. That is why entropy generation in brief form is basically a relatively important magnetic random and magnetic parameter. The variation in Bejan number due to the fluctuation of *Pr*, *Ec*, $\Omega$ and *M* is sketched in Figure 14a–d. We have seen that graphs gradually become linear from parabolic and reveal that the Bejan number inside the cylinder increases with the increment in *Pr* and *Ec* in Fig. 14a,b. In the middle region of the cylinder for $\eta = 1.6$, the irreversible phenomenon has been observed in the Bejan number. The Bejan number in the flow system decrements with the rising value of $\Omega$ as shown in Fig. 14c. It has been observed that heat transfer dominates the irreversible flow process. Therefore, the fluid friction and irreversibility of the magnetic field dominated near the stretching sheet, as can be shown. It is also noticed that when the value of $\Omega$ the *Be* declines, the influence due to heat transfer prevails far away from the stretched sheet, and for small values of $\Omega$, the influence caused by heat transfer dominates. The magnetic parameter *M* is sketched in Fig. 14d. The Bejan number decreases with increase in *M*. No influence is observed at the point $\eta$= 1.58 on *Be*. On the surface of the stretching sheet the influence of magnetic and viscous irreversibility dominates the conductive irreversibility and due to heat transfer to the upper cylinder but due to these two factors, Irreversibility equally contributes at $\eta$ = 1.58.

## Concluding remarks

In the ongoing paper, we have studied the fluid flow, Maxwell equation, and mass and heat characteristics between two concentric parallel cylinders under the variable magnetic field to see the flow behavior concerning the following emerging parameters like a magnetic parameter, squeezing parameter, Soret number, Prandtl number, magnetic Reynold’s number and heat sink/source. The proposed model formulated the fluid flow by continuity equation, momentum equation, Maxwell equation, and coupled energy and species equation with boundary conditions has been reduced into a set of highly nonlinear systems of ODEs by Lie group of similarity transformation. The proposed model is solved numerically by a highly efficient and extensively validated Parametric Continuation Method (PCM). In addition, the model outcomes and numerical scheme have been validated by solving the proposed model through another numerical system (BVP4C) in MATLAB and found a close correspondence. From the results of the model, we have drawn the following conclusion:

- The squeezing number *S* effect on the velocity profile reveals that the trend of the velocity profile is opposite and becomes parabolic.
- The temperature profile augments with augmenting values of the Prandtl number.
- The contradicting behavior between temperature and concentration profiles has been observed due to the augmenting values of the heat source parameter (Hs).
- The impact of the Schmidt and Soret number on the concentration profile reveals that the incrementing values of the Schmidt and Soret number decrements the concentration profile.
- The existence of the magnetic field reduces the magnetic profile due to the Lorentz force which slows down the movement of the fluid.

## List of symbols

*P*: Pressure
*r*, *z*: Cylindrical Coordinates
*u*: r-axis velocity
*w*: z-axis velocity
*t*: time
*Sr*: Soret number
$Re_{m}$: Rynold’s Magnetic Parameter
*Pr*: Prandtl number
$$(\rho Cp)$$: Specific Heat
*S*: Squeezing number
$b_1, b_3$: Components of Magnetic field
*Sc*: Schmidt number
*Ec*: Eckert number
$$\kappa$$: Thermal conductivity
$$\mu$$: Viscosity
$$\nu$$: Kinematic viscosity
*M*: Magnetic parameter
$$\sigma$$: Electrical conductivity
$$\rho$$: Density
*Hs*: Heat Source/Sink
*a*(*t*): Inner Cylinder
*C*1: Chemical reaction paramter
*b*(*t*): Outer Cylinder
$$\frac{T_m-T_0}{T_h-T_0}$$: Temperature difference
